# Supplementary material for: Unveiling the Bioactive Potential of the Invasive Jellyfish Phyllorhiza punctata Through Integrative Transcriptomic and Proteomic Analyses
Source: Biomolecules. 2025 Aug 4;15(8):1121. doi: 10.3390/biom15081121 (PMC12383608; doi:10.3390/biom15081121)
Supplement: Supplementary file 1 [file biomolecules-15-01121-s001.zip › Suplementary Material/FileS3/PEPFOLD4/XP_065053669_3153/clusters.html]

 PEP-FOLD clusters


Download archive of all models  here   

|  |  |  |
| --- | --- | --- |
| # | file | sOPEP |
| # cluster 1 - Size: 3 |  |  |
|  | XP\_065053669\_3153-model1 | -29.3992 |
|  | XP\_065053669\_3153-model1.1 | -25.6755 |
|  | XP\_065053669\_3153-model1.2 | -24.5388 |
| # cluster 2 - Size: 1 |  |  |
|  | XP\_065053669\_3153-model2 | -28.5778 |
| # cluster 3 - Size: 1 |  |  |
|  | XP\_065053669\_3153-model3 | -19.15 |
| # cluster 4 - Size: 1 |  |  |
|  | XP\_065053669\_3153-model4 | -18.8801 |
| # cluster 5 - Size: 1 |  |  |
|  | XP\_065053669\_3153-model5 | -17.5446 |
| # cluster 6 - Size: 11 |  |  |
|  | XP\_065053669\_3153-model6 | -17.3185 |
|  | XP\_065053669\_3153-model6.1 | -16.744 |
|  | XP\_065053669\_3153-model6.2 | -16.5653 |
|  | XP\_065053669\_3153-model6.3 | -16.4474 |
|  | XP\_065053669\_3153-model6.4 | -15.7653 |
|  | XP\_065053669\_3153-model6.5 | -14.9373 |
|  | XP\_065053669\_3153-model6.6 | -14.7968 |
|  | XP\_065053669\_3153-model6.7 | -14.2246 |
|  | XP\_065053669\_3153-model6.8 | -14.1339 |
|  | XP\_065053669\_3153-model6.9 | -13.0903 |
|  | XP\_065053669\_3153-model6.10 | -10.8133 |
| # cluster 7 - Size: 1 |  |  |
|  | XP\_065053669\_3153-model7 | -16.1071 |
| # cluster 8 - Size: 3 |  |  |
|  | XP\_065053669\_3153-model8 | -15.9251 |
|  | XP\_065053669\_3153-model8.1 | -14.6242 |
|  | XP\_065053669\_3153-model8.2 | -14.2298 |
| # cluster 9 - Size: 1 |  |  |
|  | XP\_065053669\_3153-model9 | -15.9025 |
| # cluster 10 - Size: 8 |  |  |
|  | XP\_065053669\_3153-model10 | -15.6583 |
|  | XP\_065053669\_3153-model10.1 | -15.6193 |
|  | XP\_065053669\_3153-model10.2 | -15.2936 |
|  | XP\_065053669\_3153-model10.3 | -14.1625 |
|  | XP\_065053669\_3153-model10.4 | -14.0504 |
|  | XP\_065053669\_3153-model10.5 | -13.9468 |
|  | XP\_065053669\_3153-model10.6 | -13.6017 |
|  | XP\_065053669\_3153-model10.7 | -12.9303 |
| # cluster 11 - Size: 1 |  |  |
|  | XP\_065053669\_3153-model11 | -15.5711 |
| # cluster 12 - Size: 2 |  |  |
|  | XP\_065053669\_3153-model12 | -15.4388 |
|  | XP\_065053669\_3153-model12.1 | -14.2507 |
| # cluster 13 - Size: 1 |  |  |
|  | XP\_065053669\_3153-model13 | -14.604 |
| # cluster 14 - Size: 1 |  |  |
|  | XP\_065053669\_3153-model14 | -14.5357 |
| # cluster 15 - Size: 3 |  |  |
|  | XP\_065053669\_3153-model15 | -14.5064 |
|  | XP\_065053669\_3153-model15.1 | -12.2876 |
|  | XP\_065053669\_3153-model15.2 | -11.979 |
| # cluster 16 - Size: 2 |  |  |
|  | XP\_065053669\_3153-model16 | -13.9573 |
|  | XP\_065053669\_3153-model16.1 | -12.7338 |
| # cluster 17 - Size: 4 |  |  |
|  | XP\_065053669\_3153-model17 | -13.8306 |
|  | XP\_065053669\_3153-model17.1 | -13.8259 |
|  | XP\_065053669\_3153-model17.2 | -12.5184 |
|  | XP\_065053669\_3153-model17.3 | -11.8263 |
| # cluster 18 - Size: 2 |  |  |
|  | XP\_065053669\_3153-model18 | -13.7739 |
|  | XP\_065053669\_3153-model18.1 | -11.9403 |
| # cluster 19 - Size: 1 |  |  |
|  | XP\_065053669\_3153-model19 | -13.6728 |
| # cluster 20 - Size: 1 |  |  |
|  | XP\_065053669\_3153-model20 | -13.3985 |
| # cluster 21 - Size: 1 |  |  |
|  | XP\_065053669\_3153-model21 | -13.336 |
| # cluster 22 - Size: 1 |  |  |
|  | XP\_065053669\_3153-model22 | -13.1842 |
| # cluster 23 - Size: 1 |  |  |
|  | XP\_065053669\_3153-model23 | -13.0987 |
| # cluster 24 - Size: 1 |  |  |
|  | XP\_065053669\_3153-model24 | -13.0074 |
| # cluster 25 - Size: 1 |  |  |
|  | XP\_065053669\_3153-model25 | -12.9219 |
| # cluster 26 - Size: 1 |  |  |
|  | XP\_065053669\_3153-model26 | -12.897 |
| # cluster 27 - Size: 1 |  |  |
|  | XP\_065053669\_3153-model27 | -12.6629 |
| # cluster 28 - Size: 1 |  |  |
|  | XP\_065053669\_3153-model28 | -12.5203 |
| # cluster 29 - Size: 1 |  |  |
|  | XP\_065053669\_3153-model29 | -12.4138 |
| # cluster 30 - Size: 3 |  |  |
|  | XP\_065053669\_3153-model30 | -12.1946 |
|  | XP\_065053669\_3153-model30.1 | -11.2029 |
|  | XP\_065053669\_3153-model30.2 | -9.96499 |
| # cluster 31 - Size: 1 |  |  |
|  | XP\_065053669\_3153-model31 | -11.7673 |
| # cluster 32 - Size: 5 |  |  |
|  | XP\_065053669\_3153-model32 | -11.739 |
|  | XP\_065053669\_3153-model32.1 | -11.5251 |
|  | XP\_065053669\_3153-model32.2 | -10.9883 |
|  | XP\_065053669\_3153-model32.3 | -9.88299 |
|  | XP\_065053669\_3153-model32.4 | -9.34045 |
| # cluster 33 - Size: 2 |  |  |
|  | XP\_065053669\_3153-model33 | -11.6147 |
|  | XP\_065053669\_3153-model33.1 | -11.5199 |
| # cluster 34 - Size: 1 |  |  |
|  | XP\_065053669\_3153-model34 | -11.498 |
| # cluster 35 - Size: 1 |  |  |
|  | XP\_065053669\_3153-model35 | -11.4788 |
| # cluster 36 - Size: 1 |  |  |
|  | XP\_065053669\_3153-model36 | -11.0297 |
| # cluster 37 - Size: 1 |  |  |
|  | XP\_065053669\_3153-model37 | -10.877 |
| # cluster 38 - Size: 2 |  |  |
|  | XP\_065053669\_3153-model38 | -10.8088 |
|  | XP\_065053669\_3153-model38.1 | -10.7216 |
| # cluster 39 - Size: 4 |  |  |
|  | XP\_065053669\_3153-model39 | -10.7771 |
|  | XP\_065053669\_3153-model39.1 | -10.2132 |
|  | XP\_065053669\_3153-model39.2 | -10.0445 |
|  | XP\_065053669\_3153-model39.3 | -9.68636 |
| # cluster 40 - Size: 1 |  |  |
|  | XP\_065053669\_3153-model40 | -10.7082 |
| # cluster 41 - Size: 1 |  |  |
|  | XP\_065053669\_3153-model41 | -10.388 |
| # cluster 42 - Size: 1 |  |  |
|  | XP\_065053669\_3153-model42 | -10.3372 |
| # cluster 43 - Size: 1 |  |  |
|  | XP\_065053669\_3153-model43 | -10.3146 |
| # cluster 44 - Size: 1 |  |  |
|  | XP\_065053669\_3153-model44 | -10.3041 |
| # cluster 45 - Size: 2 |  |  |
|  | XP\_065053669\_3153-model45 | -10.2658 |
|  | XP\_065053669\_3153-model45.1 | -9.61704 |
| # cluster 46 - Size: 2 |  |  |
|  | XP\_065053669\_3153-model46 | -10.2288 |
|  | XP\_065053669\_3153-model46.1 | -9.99163 |
| # cluster 47 - Size: 2 |  |  |
|  | XP\_065053669\_3153-model47 | -10.1809 |
|  | XP\_065053669\_3153-model47.1 | -9.84934 |
| # cluster 48 - Size: 2 |  |  |
|  | XP\_065053669\_3153-model48 | -10.1526 |
|  | XP\_065053669\_3153-model48.1 | -9.84946 |
| # cluster 49 - Size: 1 |  |  |
|  | XP\_065053669\_3153-model49 | -10.052 |
| # cluster 50 - Size: 1 |  |  |
|  | XP\_065053669\_3153-model50 | -10.0379 |
| # cluster 51 - Size: 1 |  |  |
|  | XP\_065053669\_3153-model51 | -10.0283 |
| # cluster 52 - Size: 1 |  |  |
|  | XP\_065053669\_3153-model52 | -9.94468 |
| # cluster 53 - Size: 2 |  |  |
|  | XP\_065053669\_3153-model53 | -9.89591 |
|  | XP\_065053669\_3153-model53.1 | -9.64474 |
| # cluster 54 - Size: 1 |  |  |
|  | XP\_065053669\_3153-model54 | -9.76309 |
| # cluster 55 - Size: 1 |  |  |
|  | XP\_065053669\_3153-model55 | -9.75922 |
|  |  |  |
